# Supplementary material for: Progesterone activation of β1-containing BK channels involves two binding sites
Source: Nat Commun. 2023 Nov 9;14:7248. doi: 10.1038/s41467-023-42827-w (PMC10636063; doi:10.1038/s41467-023-42827-w)
Supplement: Supplementary file 1 — Supplementary information [file 41467_2023_42827_MOESM1_ESM.pdf]

Supplementary Table 1. Molecular dynamics system setup description in MOE 2019.01.

min	{ ps=10 T=0 }
heat	{ ps=100 T=(10,300) r=(0.5,100) }
nvt	{ ps=50 T=300 }
npt	{ ps=50 T=300 P=100 }
prod	{ ps=250 T=300 P=100 }
algorithm	NPA
bondConstraints	'light'
dmassHMR	0
dt	0.001
rigidWater	1
startTime	0
checkpointTime	250
sample	0.1
savePosition	0
saveVelocity	0
wrapWater	0
opendbv	0
verbose	1

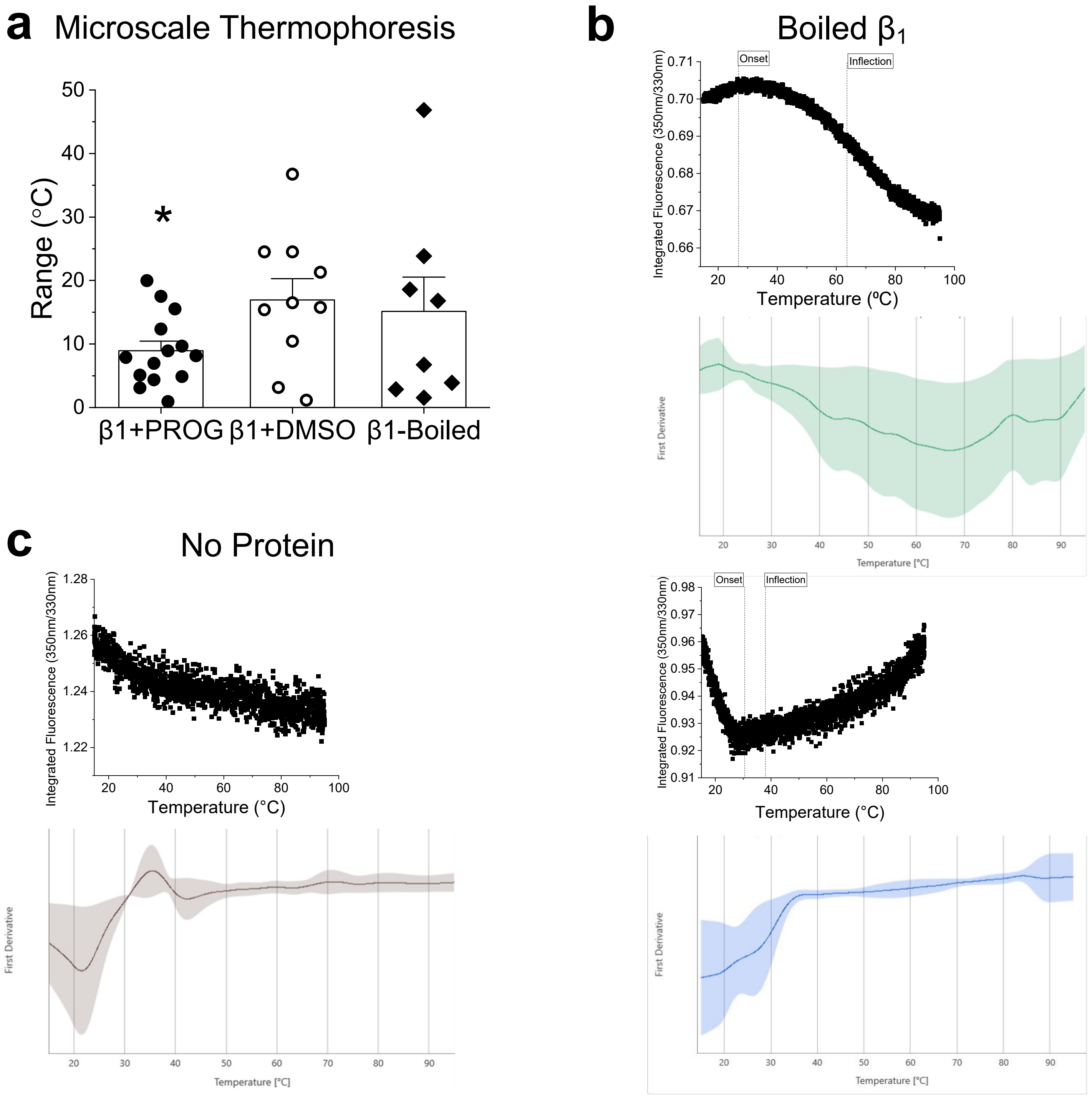

Supplementary Figure 1. Validation of Prometheus records. **a** Graphical depiction of steroid-driven, concentration-dependent changes in slo1 thermal unfolding, measured by the delta (Δ) of on-set to inflection point. *Statistically significant difference compared to its own DMSO control (P=0.036, Mann Whitney U-test, respectively), out of 6 samples, onset and inflection points could be derived from only 3 curves of boiled protein, all lacking synergism. **b** Original trace records of the thermal unfolding of β_1 in the presence of the vehicle control (DMSO) obtained from protein samples (Boiled β_1) that were heated to 95ºC for 5 min, and then allowed to cool down to room temperature prior to being loaded into Prometheus. Original traces are accompanied by images depicting high deviations in record samples, reported as the first derivative. **c** Original trace records of no protein in the presence of the vehicle control (DMSO, top). Bottom image depicts small deviations in records' first derivatives. This is in contrast to high deviations observed when protein is present and its conformational variability is exacerbated by heating/cooling cycle prior to loading into Prometheus (**b**).

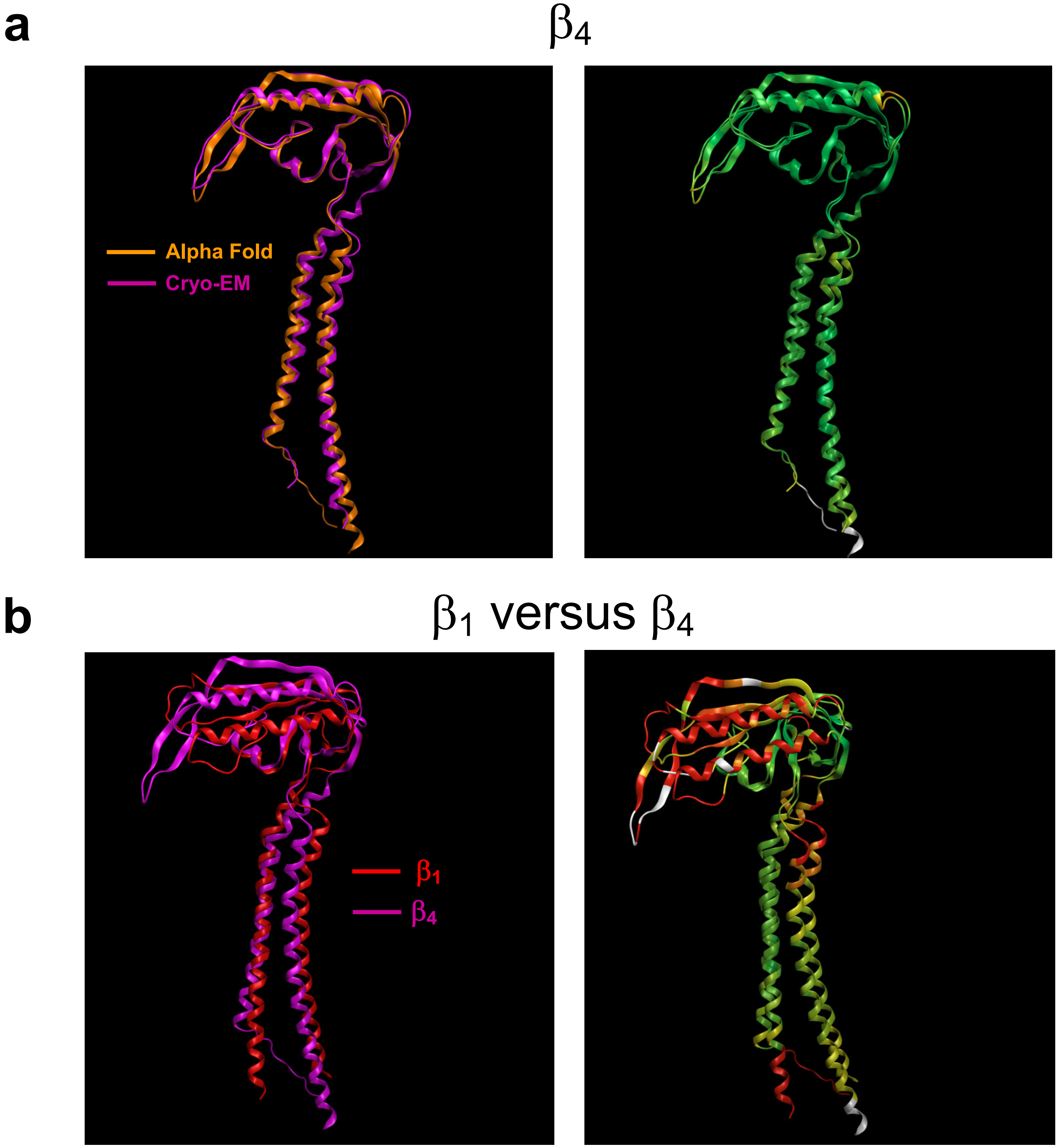

Supplementary Figure 2. Validation of β_4 and β_1 protein structures. **a** Superposition of β_4 structure obtained by Alpha Fold (orange) with structure from cryo-electron microscopy (cryo-EM; in purple)[1]; Protein Data Bank ID 6V22. The panel on the right, shows β_4 by Alpha Fold being colored according to root-mean-square deviation (RMSD) to its cryo-EM folding. Here and in **b**, green denotes a small RMSD while red reflects a large RMSD. Pairwise RMSD comparison of two structures results in averaged deviation of 1.475 Å. **b** Superposition of β_1 structure obtained by Alpha Fold (red) with β_4 structure from Alpha Fold (purple). The panel on the right shows β_1 by Alpha Fold being colored according to the RMSD of β_4. Transmembrane areas of proteins superpose with low RMSD mostly, driven by the 44% of amino acid similarity between the two proteins[2]. The largest RMSD is observed in the loop area, as the β_4 loop is longer than its β_1 counterpart[2].

Supplementary Figure 2

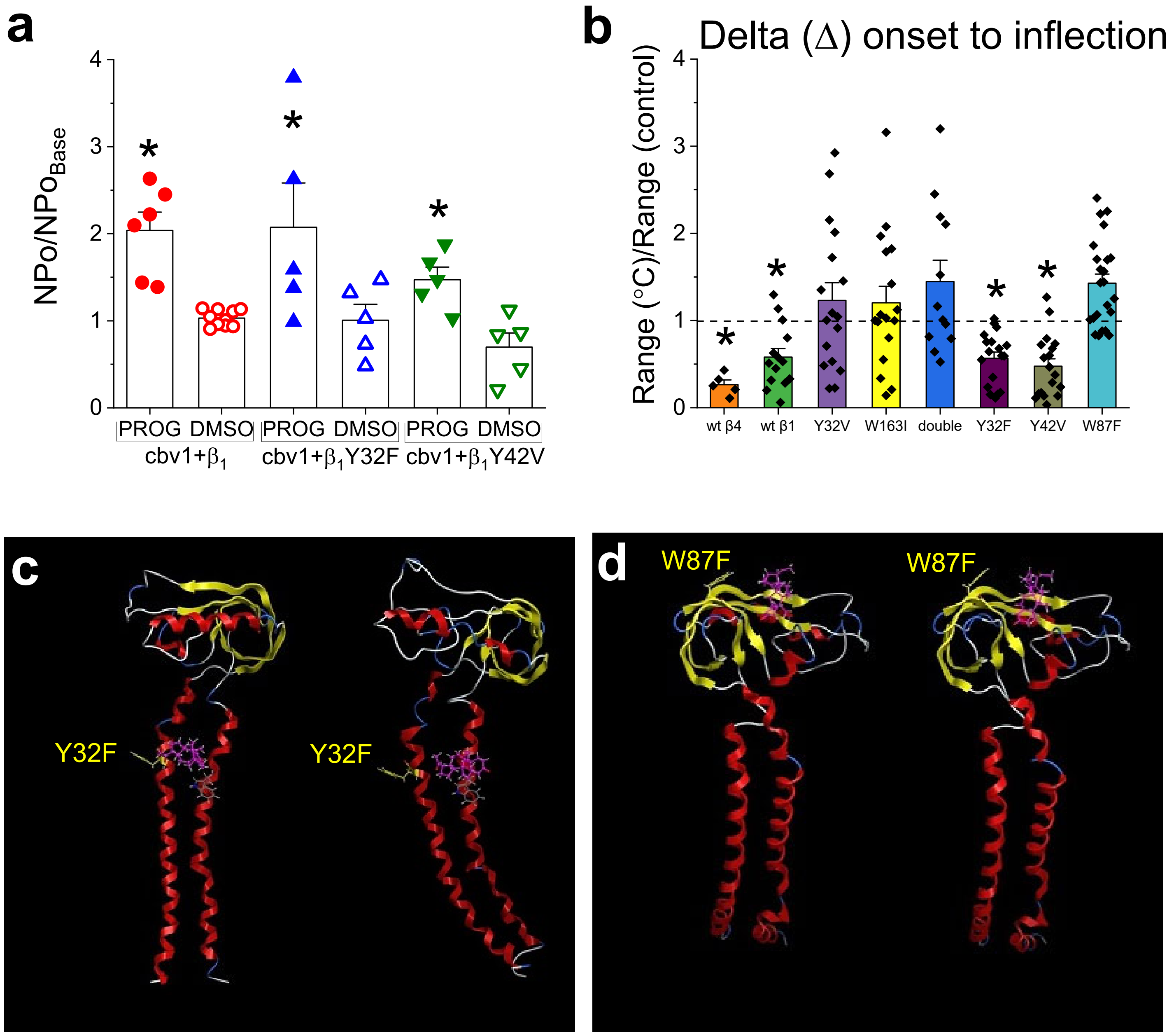

Supplementary Figure 3. Validation of mutations. **a** Bar graph depicting average changes in channel activity for the wt channel and the control mutations, β_1Y32F and β_1Y42V. Each symbol represents an individual record. *Statistically significant difference from own DMSO control. **b** Graph depiction of PROG-driven thermal unfolding, measured by the delta (Δ) of onset to inflection point. *Statistically significant difference compared to its own DMSO control ($P<0.05$, two-tailed T test). **c** Two representative snapshots of final points in the simulations for PROG interactions with β_1Y32F show that PROG, as expected, still interacts with the low affinity binding site by bridging the two TMs (left), which occurs in ~66% of the simulations. Only in ~33% of cases such bridge is not evident (right). In both models, W163 is critical for PROG-interaction with the low affinity site. **d** Two representative snapshots of final points in the simulations for PROG interactions with β_1W87F show that PROG, while hovering over the EC loop, neither gets close to the high affinity docking nor allows rotation of the two TMs as shown in main Figs. 4h,i.

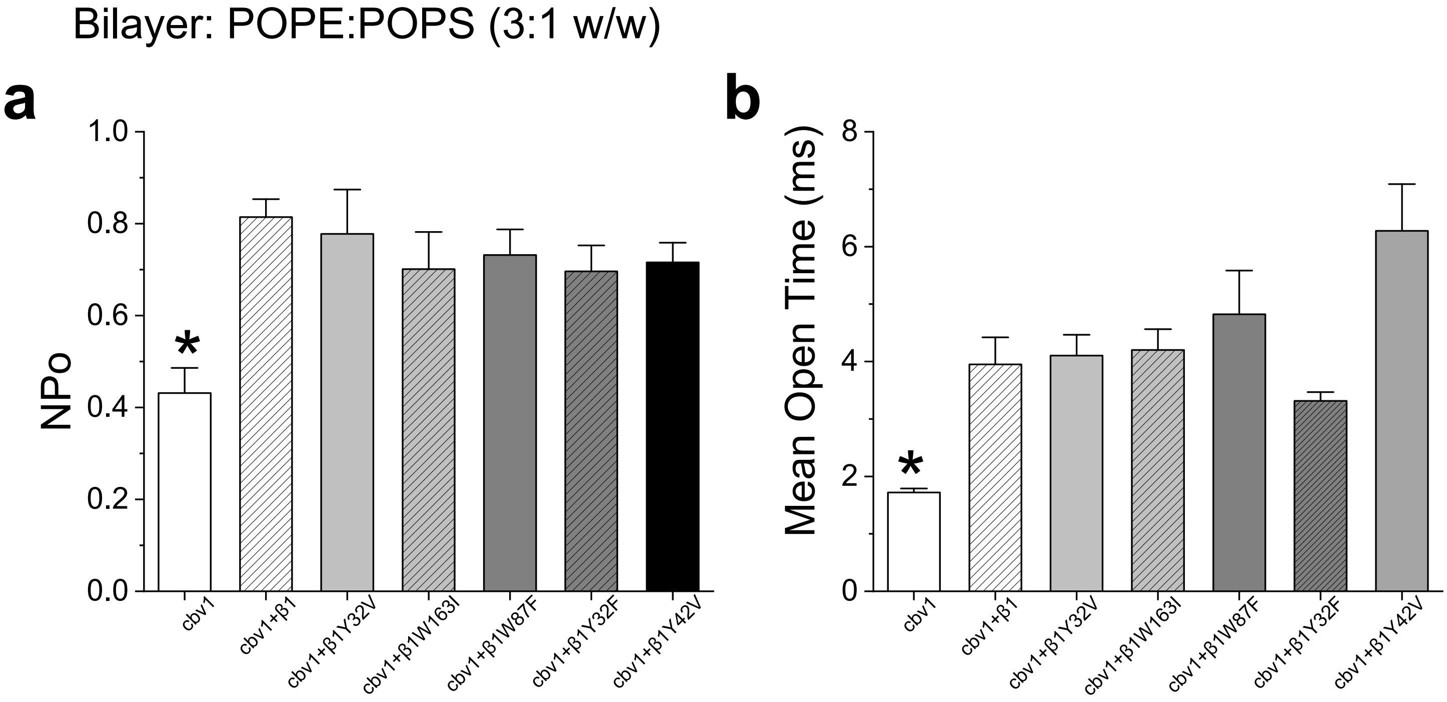

Supplementary Figure 4. Mutant association with slo1. **a** Graphical depiction of the change in average open probability (NPo) of the channel activity in absence and presence of the β_1 subunits. Slo1 (cbv1) alone is shown to have significantly smaller NPo compared with slo1, associating with the auxiliary subunit, β_1, regardless of mutations. **b** Graphical depiction of the change in the channels mean open time (ms) absence and presence of the β_1 subunits. Slo1 (cbv1) alone is shown to have significantly smaller mean open time compared with slo1, associating with the auxiliary subunit, β_1, regardless of mutations. In all experiments, channel activity was recorded at a constant voltage (0 mV), in the presence of 30 μM Ca^{2+} in the trans– and cis-solutions. * indicates statistical significance, $P<0.05$ two-tailed t-test. n=10-15

a

b

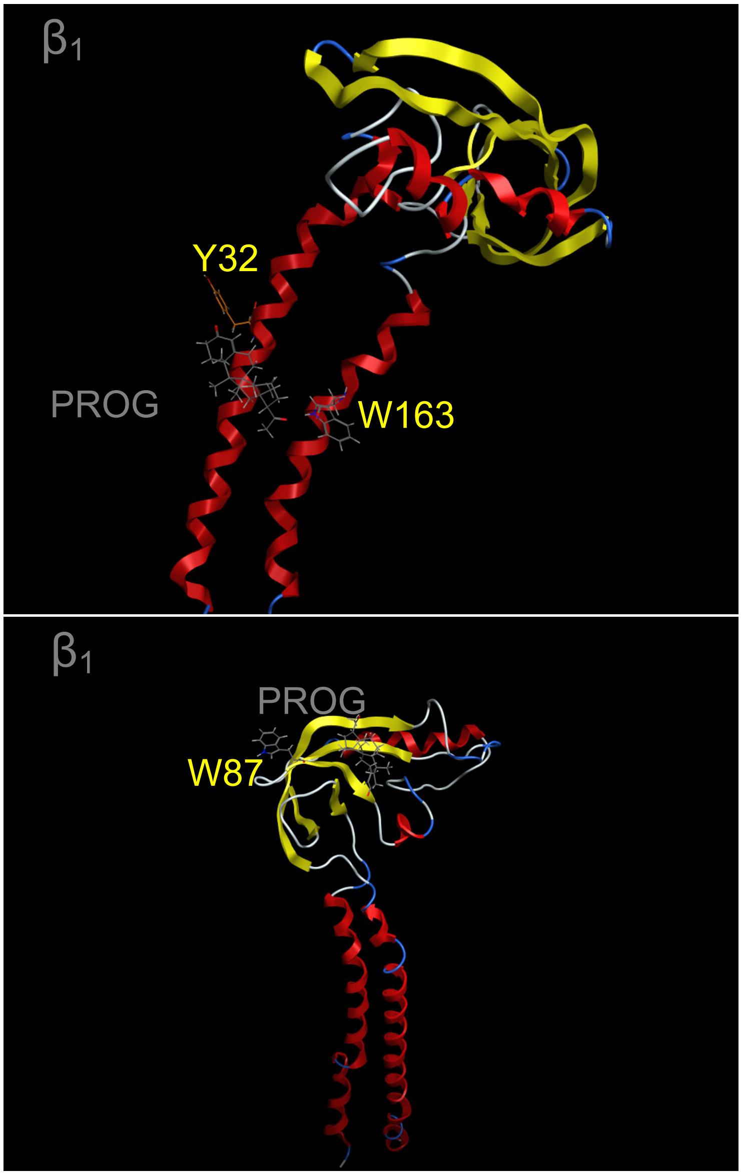

Supplementary Figure 5. Computational modeling and molecular dynamic simulations for progesterone binding to the low- and high-affinity binding sites. **a** Representative snapshot of the average starting position of molecular dynamic simulations for the docking of PROG (grey) on the β1 regulatory subunit transmembrane domains. **b** Representative snapshot of the average start position of molecular dynamic simulations for the docking of PROG (grey) on the β1 regulatory subunit loop.

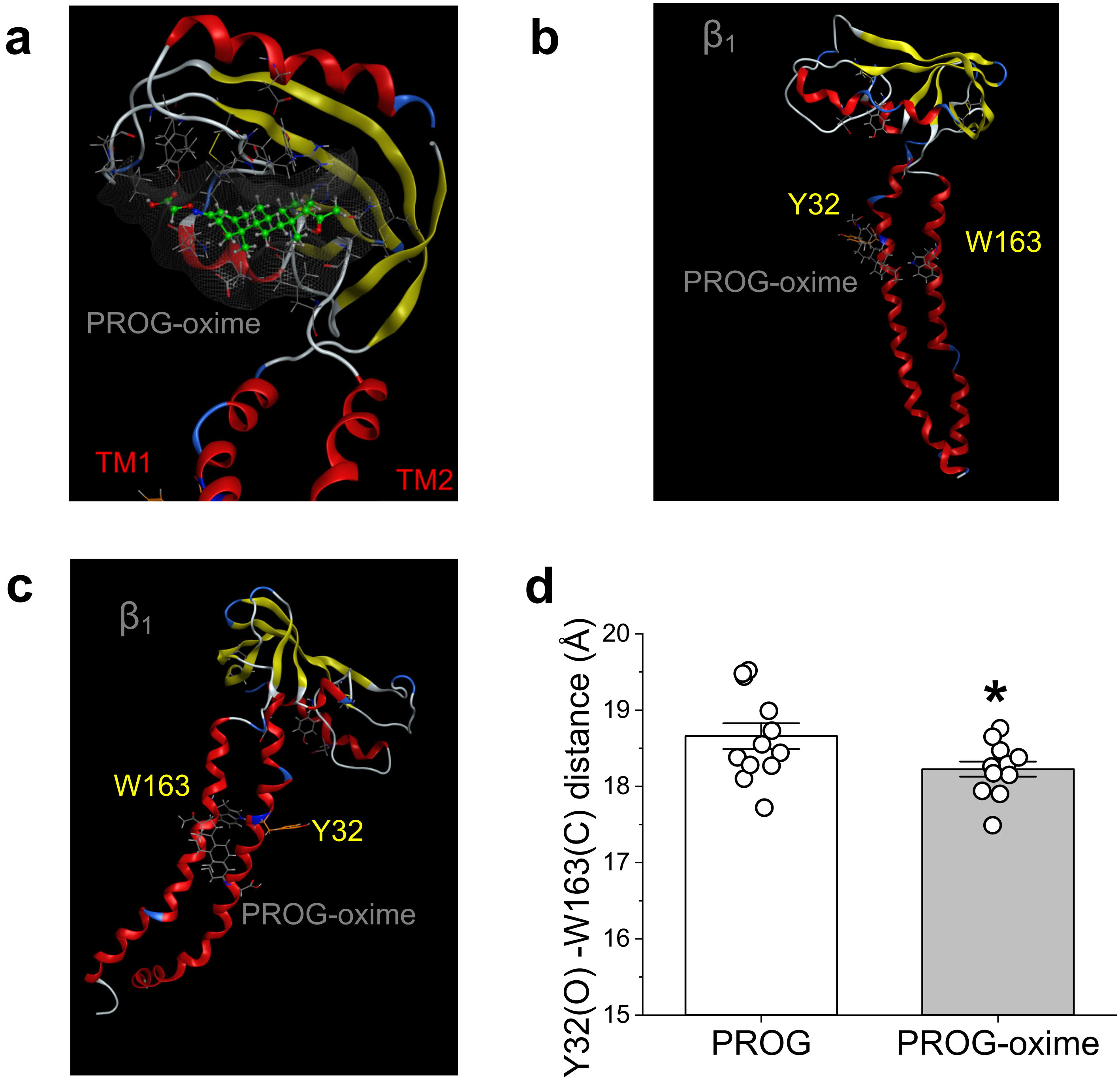

Supplementary Figure 6. Computational modeling and molecular dynamic simulations for progesterone 3-(O-carboxymethyl)oxime binding to the low-affinity site. **a** Representative snapshot of the most energetically favorable docking motif of progesterone 3-(O-carboxymethyl) oxime (green) on the β_1 regulatory subunit loop. **b** Representative snapshot of the average starting position of molecular dynamic simulations for the docking of progesterone 3-(O-carboxymethyl)oxime (grey) on the β_1 regulatory subunit transmembrane domains. **c** Representative snapshot of the average final position of molecular dynamic simulations for the docking of progesterone 3-(O-carboxymethyl)oxime (grey) on the β_1 regulatory subunit TMD. **d** Distance (Å) between oxygen of Tyr32 and backbone carbon atom of Trp163 at the production phase in the molecular dynamics simulations for PROG versus PROG-oxime on the β_1 regulatory subunit TMD. Four molecular dynamics simulations were performed for each steroid. In each simulation, distances were measured at 150, 200 and 250 ps of production phase and included into data-sets as individual points. Thus, each data-set contains 12 points. *Statistically significant difference when compared to PROG by two-tail unpaired *t*-test, p=0.0379.

Supplementary Figure 6

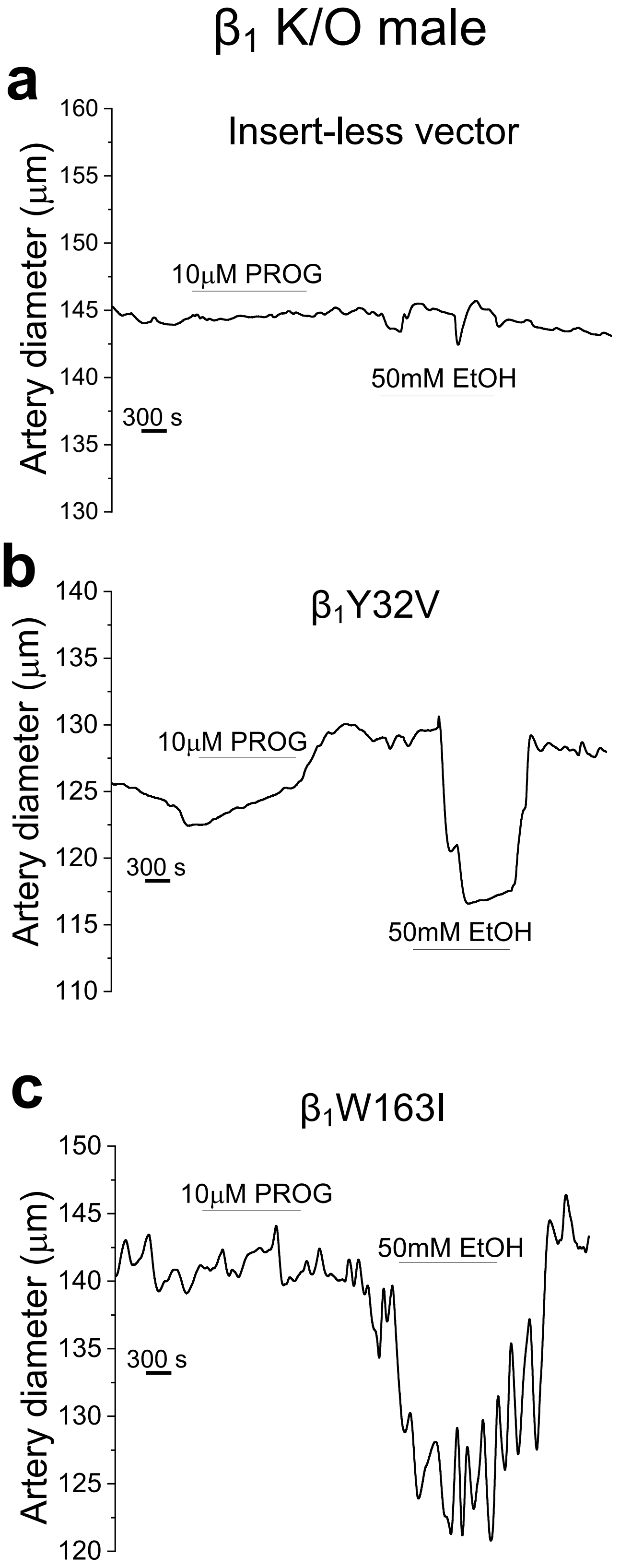

Supplementary Figure 7. BK β_1 subunit amino acid substitutions that interfere with progesterone binding prevent this steroid from counteracting alcohol-induced cerebral artery constriction, an alcohol action known to result in brain ischemia in several species, including humans[3-7]. **a-c** depict original traces of arterial diameter of de-endothelialized MCA segments from male β_1 K/O mice that were electroporated with either wt β_1 cDNA, as positive control, or β_1 constructs encoding either the Y32V or W163I substitutions within the progesterone-sensing functional site. While these mutants fail to support MCA dilation by 10 μM progesterone, they remain sensitive to alcohol-induced constriction. Arteries were in vitro pressurized at 60 mmHg prior to drug testing.

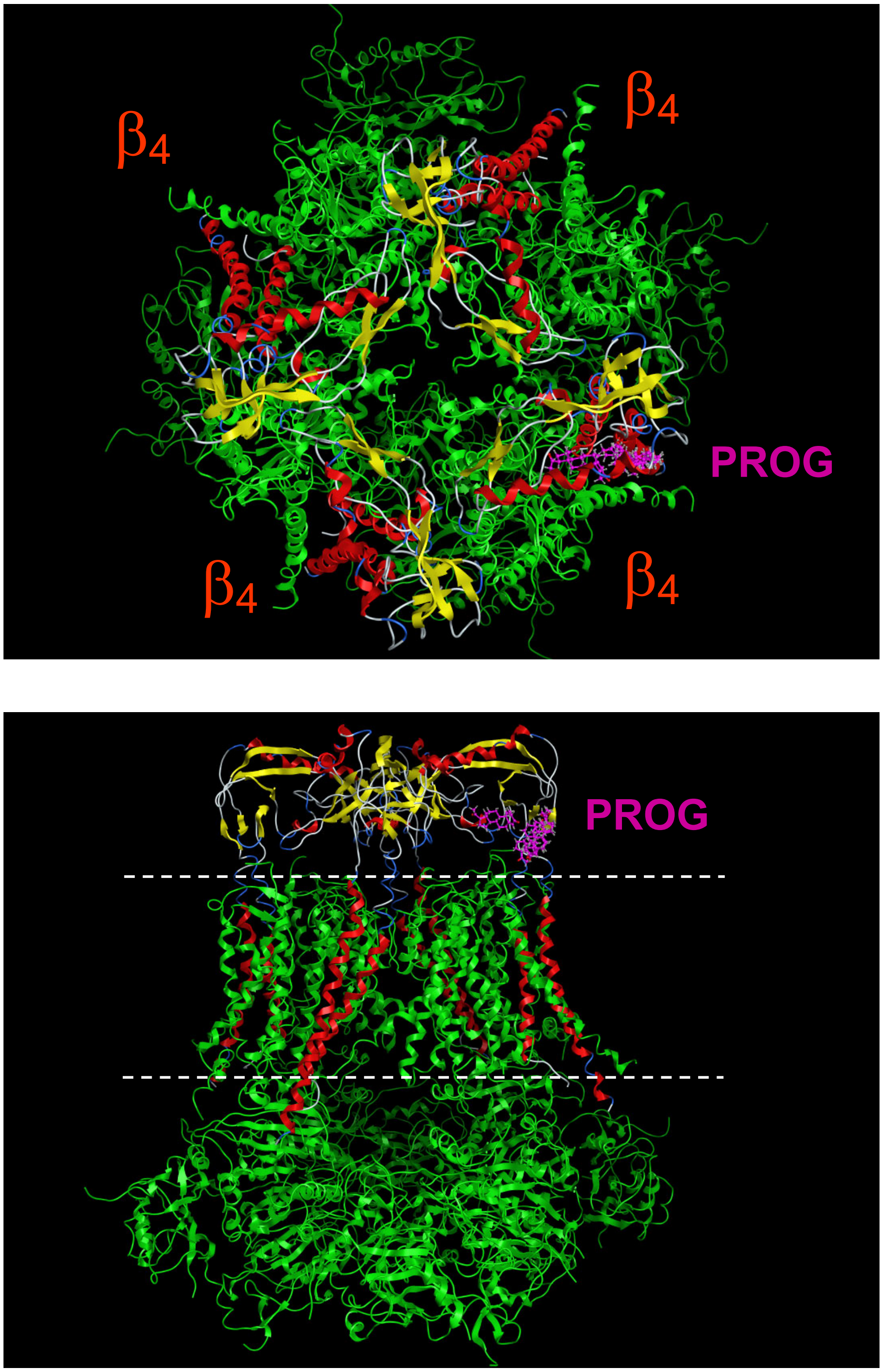

Supplementary Figure 8. PROG binding to its high-affinity site in β_4 subunits within the BK channel heterotetramer. Top (top panel) and side (bottom panel) views of a BK channel hetero-tetrameric assembly obtained from published[1] coordinates of cryo-EM BK channel complexes including β_4 (PDB 6V22). BK β_4 subunits are color-coded with red depicting alpha-helixes, yellow showing beta-sheets, and grey/blue reflecting disorganized domains and turns, respectively. BK α subunits are shown in green. Dashed horizontal lines point at membrane boundaries. Progesterone (PROG) docks to β_4 subunit loop area, as exemplified by purple color of progesterone chemical structure. Docking was performed using built-in function in MOE2022 software. Five docking poses for individual β_4 were returned, all included into the figure. Progesterone docks between key structural elements of the β_4 loop, while interface between neighboring β_4 subunits contains disorganized protein domains. These disordered areas are expected to have high degree of spatial flexibility, thus likely favoring the transduction of the conformational changes triggered by progesterone molecules binding to their sites into modification of BK channel gating and activity.

Supplementary Figure 8

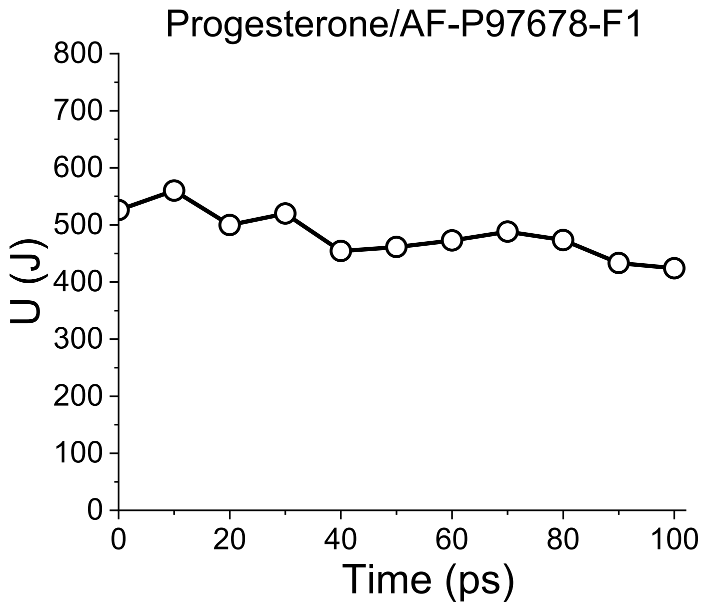

Supplementary Figure 9. Validation of molecular dynamics simulation equilibration time. Representative trace showing changes in potential energy (U) as a function of time (ps) during equilibration phase of progesterone complex with AF-P97678-F1 Model of β_1 subunit. Energy values at corresponding standard deviations were collected every 10 ps during equilibration step which lasted 100 ps. In this step, 50 ps were run using the canonical constant number of particles, volume, and temperature (nvt) ensemble. Next 50 ps were equilibrated using the isobaric-isothermal (constant number of particles, pressure, and temperature, npt) ensemble. As time passes by, U exhibits less variability.

Supplementary Information References

1. X. Tao, R. MacKinnon, Molecular structures of the human Slo1 K^+ channel in complex with β_4. *Elife* **8**, e51409 (2019).

2. R. Brenner, T. J. Jegla, A. Wickenden, Y. Liu, R. W. Aldrich, Cloning and functional characterization of novel large conductance calcium-activated potassium channel beta subunits, hKCNMB3 and hKCNMB4. *J Biol Chem* **275**, 6453-61.

3. B. M. Altura, B. T. Altura, Alcohol, the cerebral circulation and strokes, *Alcohol.* **1**, 25-31 (1982).

4. N. D. Volkow, N. Mullani, L. Gould, S. S. Adler, R. W. Guynn, J. E. Overall, S. Dewey, Effects of acute alcohol intoxication on cerebral blood flow measured with PET. *Psychiatry Res* **24**, 201-9 (1988).

5. P. Liu, Q. Xi, A. Ahmed, J. H. Jaggar, A. M. Dopico, Essential role for smooth muscle BK channels in alcohol-induced cerebrovascular constriction. *Proc Natl Acad Sci U S A*, **101**, 18217-22 (2004).

6. E. V. Sullivan, Q. Zhao, K.M. Pohl, N.M. Zahr, A. Pfefferbaum, Attenuated cerebral blood flow in fronto-limbic and insular cortices in Alcohol Use Disorder: Relation to working memory. *J Psychiatr Res* **136**, 140–8 (2021).

7. S. Mysiewicz, K. C. North, L. Moreira, Jr., S. J. Odum, A. N. Bukiya, A. M. Dopico, Interspecies and regional variability of alcohol action on large cerebral arteries: regulation by KCNMB1 proteins. *Am J Physiol*
