# Supplementary material for: Progesterone activation of β1-containing BK channels involves two binding sites
Source: Nat Commun. 2023 Nov 9;14:7248. doi: 10.1038/s41467-023-42827-w (PMC10636063; doi:10.1038/s41467-023-42827-w)
Supplement: Supplementary file 3 — Reporting Summary [file 41467_2023_42827_MOESM3_ESM.pdf]

nature portfolio

Corresponding author(s): Alex Dopico

Last updated by author(s): October 18, 2023

Reporting Summary

Nature Portfolio wishes to improve the reproducibility of the work that we publish. This form provides structure for consistency and transparency in reporting. For further information on Nature Portfolio policies, see our Editorial Policies and the Editorial Policy Checklist.

Statistics

For all statistical analyses, confirm that the following items are present in the figure legend, table legend, main text, or Methods section.

n/a	Confirmed	
☐	☒	The exact sample size (*n*) for each experimental group/condition, given as a discrete number and unit of measurement
☐	☒	A statement on whether measurements were taken from distinct samples or whether the same sample was measured repeatedly
☐	☒	The statistical test(s) used AND whether they are one- or two-sided<br>*Only common tests should be described solely by name; describe more complex techniques in the Methods section.*
☒	☐	A description of all covariates tested
☐	☒	A description of any assumptions or corrections, such as tests of normality and adjustment for multiple comparisons
☐	☒	A full description of the statistical parameters including central tendency (e.g. means) or other basic estimates (e.g. regression coefficient) AND variation (e.g. standard deviation) or associated estimates of uncertainty (e.g. confidence intervals)
☐	☒	For null hypothesis testing, the test statistic (e.g. *F*, *t*, *r*) with confidence intervals, effect sizes, degrees of freedom and *P* value noted<br>*Give P values as exact values whenever suitable.*
☒	☐	For Bayesian analysis, information on the choice of priors and Markov chain Monte Carlo settings
☒	☐	For hierarchical and complex designs, identification of the appropriate level for tests and full reporting of outcomes
☒	☐	Estimates of effect sizes (e.g. Cohen's *d*, Pearson's *r*), indicating how they were calculated

Our web collection on statistics for biologists contains articles on many of the points above.

Software and code

Policy information about availability of computer code

Data collection	pClamp8 and pClamp11 software for electrophysiological data acquisition; Nanotemper PR.therm-control software for thermophoresis data acquisition; MOE 2019.01 Suite for computational docking and stimulation data acquistion; IonWizard software for artery diameter data acquisition.
Data analysis	pClamp10.7 software for electrophysiological data analysis; Nanotemper PR.therm-control sofware for thermophoresis data analysis; Origin 2020 software for thermophoresis, electrophysiology, and vessel diameter data analysis and final plotting; MOE 2029.01 Suite for computational docking and stimulation data analysis; IonWizard software for artery diameter data analysis; InStat 3.05 software for statistical data analysis.

For manuscripts utilizing custom algorithms or software that are central to the research but not yet described in published literature, software must be made available to editors and reviewers. We strongly encourage code deposition in a community repository (e.g. GitHub). See the Nature Portfolio guidelines for submitting code & software for further information.

Data

Policy information about availability of data

All manuscripts must include a data availability statement. This statement should provide the following information, where applicable:

- Accession codes, unique identifiers, or web links for publicly available datasets
- A description of any restrictions on data availability
- For clinical datasets or third party data, please ensure that the statement adheres to our policy

slo1 (cbv1 isoform): AY330293; KCNMB1 product: NP_011452, FJ154955; KCNMB4 product: NP_055320, AY028605; KCNMB1 protein structure: AF-P97678-F1 Model, PDB P97678; KCNMB4 protein structure: AF-Q9ESK8-F1 Model, PDB: Q9ESK8.

Human research participants

Policy information about studies involving human research participants and Sex and Gender in Research.

Reporting on sex and gender	N/A
Population characteristics	N/A.
Recruitment	N/A.
Ethics oversight	N/A.

Note that full information on the approval of the study protocol must also be provided in the manuscript.

Field-specific reporting

Please select the one below that is the best fit for your research. If you are not sure, read the appropriate sections before making your selection.

[x] Life sciences [ ] Behavioural & social sciences [ ] Ecological, evolutionary & environmental sciences

For a reference copy of the document with all sections, see nature.com/documents/nr-reporting-summary-flat.pdf

Life sciences study design

All studies must disclose on these points even when the disclosure is negative.

Sample size	Sample sizes were based on our previous experience with specific experiment type. We also utilized https://www.dssresearch.com/knowledgecenter/toolkitcalculators/statisticalpowercalculators.aspx to calculate sample size for power analysis. An α error of confidence level was set at 0.05. With conservative estimation of effect size=10% and observed deviations, n=8 gives power of 81%, which renders the indicated minimum.
Data exclusions	When number of observations did not exceed 10, no data were excluded. For groups with number of data-points exceeding 10, we used Outlier detection option in Origin 2022 software which bases outlier detection on Gaussian distributions.
Replication	It is a laboratory policy to repeat each experiment on at least three independent occasions. Data reported in this manuscript were reproducible.
Randomization	Distribution of animals and cerebral arteries was random. We either use randomizations software, or a simple raffle drawing in the lab to ensure randomization.
Blinding	Whenever possible, we practice blind approach to data acquisition and analysis.

Reporting for specific materials, systems and methods

We require information from authors about some types of materials, experimental systems and methods used in many studies. Here, indicate whether each material, system or method listed is relevant to your study. If you are not sure if a list item applies to your research, read the appropriate section before selecting a response.

Materials & experimental systems

n/a	Involved in the study	
[ ]	[x]	Antibodies
[ ]	[x]	Eukaryotic cell lines
[x]	[ ]	Palaeontology and archaeology
[ ]	[x]	Animals and other organisms
[x]	[ ]	Clinical data
[x]	[ ]	Dual use research of concern

Methods

n/a	Involved in the study	
[x]	[ ]	ChIP-seq
[x]	[ ]	Flow cytometry
[x]	[ ]	MRI-based neuroimaging

Antibodies

Antibodies used	Rabbit polyclonal anti-flag antibody (10 µg; Abcam, Cat# ab205606, RRID:AB_2916341) was cross-linked with dynabeads for immunoprecipitation of relevant flag-tagged proteins.
Validation	We performed negative control experiments; flag-tagged protein was precipitated on a dynabeads column without antibody. Eluates from antibody-containing and antibody-lacking dynabeads columns were run on Western blot to confirm the presence of the band for correct size of our protein in the former but not in the latter.

Eukaryotic cell lines

Policy information about cell lines and Sex and Gender in Research

Cell line source(s)	Chinese hamster ovary (CHO) cells were purchased from Sigma-Aldrich (85051005).
Authentication	We did not perform in-depth authentication of our CHO cell line as it was merely used for protein expression. However, we did validate with electrophysiology that our non-transfected cells lack detectable BK currents.
Mycoplasma contamination	Cell line was not tested for mycoplasma contamination.
Commonly misidentified lines (See ICLAC register)	None.

Animals and other research organisms

Policy information about studies involving animals; ARRIVE guidelines recommended for reporting animal research, and Sex and Gender in Research

Laboratory animals	Study involved C57BL/6J animals and KCNMB1 global knock-outs on C57BL/6J background, 8-12 weeks old.
Wild animals	Study did not involve wild animals.
Reporting on sex	Both male and female mice were used in the study.
Field-collected samples	Study did not involve sample collections from the field.
Ethics oversight	The care of animals and experimental protocols were reviewed and approved by the Institutional Animal Care and Use Committee of the University of Tennessee Health Science Center, which is an institution accredited by the Association for Assessment and Accreditation of Laboratory Animal Care international (AAALACi).

Note that full information on the approval of the study protocol must also be provided in the manuscript.
